# Perspectives of healthcare professionals on cross-sectoral collaboration between mental health centers and municipalities: A qualitative study

**DOI:** 10.1177/00207640241299387

**Published:** 2024-12-07

**Authors:** Kim Jørgensen, Rikke Juhl, Morten Hansen, Birgitte Lerbæk, Jesper Frederiksen, Emma Watson, Merete Bjerrum, Bengt Karlsson

**Affiliations:** 1 Faculty of Health Sciences, Department of Nursing and Health Promotion, Oslo Metropolitan University, Norway; 2 Department of People and Technology, Roskilde University, Denmark; 3 Specialized Nurse in Psychiatric and Child Psychiatric Nursing, Psychiatry Region Zealand, Roskilde, Denmark; 4 Bostedsteamet, Ishøj, Kobenhavn O, Denmark; 5 Unit for Psychiatric Research, Psychiatry, Aalborg University Hospital, Denmark; 6 Zealand University Hospital, Medical Department, Roskilde, Denmark; 7 Department of Regional Health Research, University of Southern Denmark, Odense, Denmark; 8 Nottinghamshire Healthcare NHS Foundation Trust, Nottingham, UK; 9 Research Unit of Nursing and Healthcare, Institute of Public Health, Aarhus University, Denmark; 10 The Centre of Clinical Guidelines, Department of Clinical Medicine, Aalborg University, Denmark; 11 Department of Health, Social, and Welfare Studies, Faculty of Health and Social Sciences, University of Southeastern Norway, Drammen, Norway

**Keywords:** Collaboration, healthcare professionals, interdisciplinary, mental health, municipalities, nursing, patient-centered care, qualitative research, service delivery, user involvement

## Abstract

**Aims:**

This paper aims to explore the intricacies of cross-sectoral collaboration in mental health care, focusing on the perspectives of health professionals across various disciplines. It seeks to understand how collaboration can enhance service delivery and patient outcomes while identifying existing challenges.

**Background:**

The evolving healthcare landscape emphasizes the importance of integrating services across sectors, particularly in mental health care, to improve continuity and efficiency of care.

**Design:**

The study utilizes qualitative methods to investigate health professionals’ experiences with cross-sectoral collaboration in mental health services.

**Methods:**

Purposive sampling was used to select 21 health professionals for focus group discussions held at a mental health center in Region Zealand, Denmark. Data was collected through these discussions, and content analysis was performed to extract key themes. The data collection took place in 2022.

**Results/findings:**

Health professionals recognized the potential benefits of cross-sectoral collaboration, such as enhanced service delivery and improved patient outcomes. However, significant barriers were identified, including bureaucratic obstacles, communication gaps, and insufficient organizational support.

**Conclusion:**

Cross-sectoral collaboration in mental health care offers significant advantages, but challenges must be addressed to fully realize its potential. Efforts should focus on policy reforms, organizational support, and fostering interdisciplinary communication to improve care delivery.

## Introduction

In recent years, cross-sectoral collaboration in mental health care has garnered increasing attention, driven by the recognition of its potential to provide more cohesive and integrated care. This approach aligns with national healthcare policies and is supported by various stakeholders aiming to improve service delivery for individuals using mental health services (Jørgensen, Lerbæk, et al., 2024; Sangill et al., 2019; Viksveen et al., 2022). By bridging different sectors, cross-sectoral collaboration addresses the complex needs of service users, offering a more holistic approach to care (Jørgensen, Hansen, et al., 2024). However, despite its potential, research on how professionals from various healthcare sectors perceive and engage in such collaboration remains limited (Castro et al., 2016; Jørgensen et al., 2021). This study aims to fill that gap by examining the perspectives, challenges, and goals of healthcare professionals (HP), exploring both the barriers and facilitators to effective cross-sectoral collaboration in mental health care. Through this analysis, the research seeks to contribute to the development of more integrated and efficient care models that better serve mental health service users.

## Background

Cross-sectoral collaboration in hospitals and municipal agencies is essential in the context of mental health care, as it involves the coordinated effort of various stakeholders, including service users (SU) their careers and loved ones, HP, and municipal agencies, to participate in the decision-making processes concerning treatment and care. This collaborative approach is particularly critical in mental health settings, where integrating services across sectors can significantly enhance care continuity, ensure that treatment is tailored to the diverse needs of individuals, and address complex mental health challenges (Bowersox et al., 2013; Leemeijer & Trappenburg, 2016; Oxelmark et al., 2018; Söderberg et al., 2022). By actively involving all relevant parties in the treatment process, mental HP can transition from a paternalistic model to a more collaborative and inclusive framework, where the voices and preferences of SU and other stakeholders are acknowledged and valued (Biringer et al., 2017; Davidson, 2016; Ness et al., 2014). This engagement fosters a therapeutic alliance built on trust and respect, enhancing communication and understanding of the unique needs and situations of each SU (Coulter et al., 2011; Hain & Sandy, 2013; Souraya et al., 2018). Furthermore, cross-sectoral collaboration allows for care that is tailored to the individual’s holistic well-being, accommodating their diverse and complex mental health challenges through interventions that are culturally, socially, and personally relevant.

The role of HP in facilitating cross-sectoral collaboration is crucial. As frontline mediators, they ensure that all parties, including SU, are encouraged and supported to play an active role in the collaborative care process. Current research on the perspectives of HP, SU, and municipal agencies on this topic is limited (Jansen & Hanssen, 2017; Leemeijer & Trappenburg, 2016; Oxelmark et al., 2018). Understanding these perspectives enables us to consider the barriers and challenges for professionals working across sectors. Their first hand experience of navigating the fault lines and gaps between sectors will inform the development of innovative approaches, that prioritize cross-sector collaboration and ultimately support the recovery process. This study aims to fill this knowledge gap by exploring the attitudes, challenges, and aspirations related to cross-sectoral collaboration from the viewpoints of HPs across various disciplines. By doing so, it seeks to enhance the integration of comprehensive and coordinated care strategies into mental health treatment planning.

## Method

This section outlines the approach taken to explore healthcare professionals’ involvement in cross-sectoral collaboration within mental health services. Recognizing the complexity of mental healthcare provision, our methodology was designed to capture a wide range of perspectives and the nuanced dynamics of cross-sector collaboration in supporting individuals during their recovery process.

## Sample

To gain a comprehensive understanding of HPs’ involvement in cross-sectoral collaboration within mental health services, purposive sampling was utilized to ensure a diverse and representative selection of participants (Ames et al., 2019). This approach deliberately included professionals from various backgrounds and settings, such as nurses, assistant nurses, occupational therapists, peer workers, pedagogs, psychomotor therapist and social workers, working in both inpatient wards (closed and open) and outpatient units. The study was conducted at a major mental health center in Region Zealand, Denmark, encompassing three inpatient wards and two outpatient departments. Detailed explanations about the study were provided during individual meetings with each group. A total of 21 participants provided informed consent and took part in the study (see Table 1).

**Table 1.** Focus groups.

Context	Number of participations	Title
An acute mental health unit	5	Three nurses and two ass Nurses
An acute mental health unit	2	A nurse and an assistant Nurse
An acute mental health unit	5	Three nurses, an ass nurse, and occupational therapist
An outpatient unit	4	Two nurses, a peer worker, and a pedagog
An outpatient unit	5	Two nurses, a social worker, an occupational therapist, and psychomotor therapist
The number of participants	21	

The participants included 3 men and 18 women with an average age of 46 years. Most were registered nurses (RNs) with professional experience ranging from 5 to 25 years. Some participants had received specialized training in psychiatric nursing.

The research team comprised eight members with diverse professional backgrounds, including two individuals with lived experiences of mental health challenges. It consisted of six RNs, one educator, two peer supporters, and six individuals holding PhDs. The team was geographically diverse, drawing on expertise from various international locations, and employed qualitative methods to investigate mental healthcare practices, including facilitating focus group interviews. The primary authors led these interviews in conjunction with other team members.

## Focus group interviews

Focus group were conducted within each mental health ward and outpatient department, engaging distinct groups (see Table 1). This method facilitated the capture of nuanced participant experiences, resulting in rich and high-quality data (Morgan, 2012, 2014, 2019) Our approach encouraged dynamic discussions on cross-sectoral collaboration in mental health centers and with municipalities. Drawing on existing literature (Olasoji et al., 2018; Waters et al., 2015), we formulated questions to explore participants’ experiences with cross-sectoral collaboration within mental health services. Using this question list, we initiated discussions with broad inquiries, followed by specific probes to elicit detailed responses (see Table 2). We emphasized collaborative dialog based on the provided questions and highlighted the distinction between focus groups and individual interviews. To foster interaction, we utilized active listening techniques and encouraged participation through both verbal and nonverbal cues.

**Table 2.** Interview guide.

Theme	Research questions	Interview questions
Uncover the participants’ experience	Profession, years of experience in psychiatry Identify which sector participants are employed in.	We’ll start with a round. Can you begin by telling us your age, profession, and years of experience in psychiatry? Where is your current employment?
Cross-Sector Collaboration Between Mental Health Centers and Municipalities	How can cross-sector collaboration between mental health centers and municipalities be improved to better support the user recovery process	What value do you place on cross-sector collaboration? How do you incorporate collaboration and coordination in your daily work? Can you provide an example of how you have supported cross-sector collaboration to better support the user recovery process?
Adaptation of Cross-Sector Collaboration	How can cross-sector collaboration be adapted to better meet the needs of patients and improve overall care outcomes	In what ways do you have the opportunity to tailor the course to suit the patient’s needs? What factors might limit the ability to consider individual preferences? Can you provide an example where you were unable to consider individual preferences, even though it would have been desirable?
ake a summary	What specific changes or improvements would you suggest to enhance cross-sector collaboration between mental health centers and municipalities in order to better support the user recovery process	What specific strategies or initiatives do you believe could improve communication and coordination between mental health centers and municipalities? Can you identify any current barriers to effective cross-sector collaboration and suggest practical solutions to overcome them? In your experience, what role do patients and their families play in enhancing cross-sector collaboration, and how can their involvement be better facilitated?

## Data analysis

Data analysis utilized inductive content analysis, conducted through a structured four-stage approach (Elo, 2007; Erlingsson & Brysiewicz, 2017; Graneheim & Lundman, 2004; Vears et al., 2022). Initially, transcripts were comprehensively reviewed to gain a holistic understanding. Meaningful units, representing statements relevant to the research objective, were identified and extracted using specific analytical questions. These units were then analyzed to identify common categories, which were summarized into five descriptive categories. A cross-sectional analysis was subsequently conducted to explore patterns of consistency and divergence, leading to the identification of an overarching explanatory category and four distinct categories. Collaboration within the research team facilitated these analytical steps (see Table 3). NVivo software was utilized to organize the analytical process.

**Table 3.** Meaning units and themes.

Analysis questions	Meaning units	Themes	Main theme
1. What are the main challenges you face with cross-sector collaboration between mental health centers and municipalities, and what strategies have you found effective in overcoming these challenges? 2. How can cross-sector collaboration between mental health centers and municipalities be improved to better support the user recovery process? 3. How do you incorporate collaboration and coordination in your daily work? Can you provide an example of how you have supported cross-sector collaboration to better support the user recovery process? 4. What specific strategies or initiatives do you believe could improve communication and coordination between mental health centers and municipalities? Can you identify any current barriers to effective cross-sector collaboration?	Healthcare professionals face significant obstacles due to rigid legislative and bureaucratic boundaries between institutions and sectors in mental health care. These challenges disrupt care continuity and hinder effective collaboration, leading to fragmented service delivery. This theme highlights the need for policy reforms to improve integration and coordination across healthcare sectors.	Challenges of Institutional and Sectoral Boundaries	Enhancing Integrated and Collaborative Mental Health Care through Overcoming Systemic Barriers and Promoting Patient-Centered Approaches
	User-Centered Care and Inclusion emphasizes involving service users early in their care, respecting their unique needs and preferences. This approach builds trust, personalizes treatment, and ensures better health outcomes through continuous collaboration.	Importance of User-Centered Care and Inclusion	
	The importance of interdisciplinary collaboration and clear role understanding emerged as a key theme. Participants stressed that clear role definitions across disciplines like nursing, social work, and psychiatry are crucial for effective, patient-centered care. This clarity helps prevent overlap, reduce conflicts, and ensures mutual respect, fostering a productive team environment.	Interdisciplinary Collaboration and Role Understanding	
	In the focus group interviews, healthcare professionals emphasized the importance of involving service users in treatment planning. Empowering users to actively participate in decision-making fosters ownership of their care, leading to more personalized and effective outcomes. Transparency and open dialog were highlighted as key factors in enhancing user satisfaction and improving health outcomes.	Empowerment and Ownership in Treatment Planning	
	Focus group interviews with nurses and health professionals revealed that experience and ongoing professional development are vital for effective collaboration in mental health care. Participants emphasized that adapting to various care settings and understanding different roles within the healthcare system are crucial for delivering patient-centered care. This theme highlights the need for continuous learning and professional growth to improve the quality of mental health services.	Experience and Professional Development	

## Ethical considerations

The research adhered to ethical standards in scientific inquiry, receiving approval from the Research Ethics Committee (Institutional Review Board) at Roskilde University, Denmark under reference number KJ-03.23. The study complied with the principles outlined in the Helsinki Declaration (The Ministry of Interior and Health, 2017; World Medical Association, 2013). As the research did not aim to exert physical or psychological influence on participants, formal permission from a biomedical ethics committee was deemed unnecessary. Participants were fully briefed on the project and provided both written and verbal consent to participate. The principal investigator elucidated the study’s objectives and ensured participants comprehended their legal and ethical rights. Approval to collect empirical data was obtained from a manager at the Regional Zeeland Denmark mental health organization. All invited participants willingly consented, and there were no withdrawals from the study. The research team ensured data confidentiality and security by anonymizing personal information and securely storing data in compliance with GDPR and institutional guidelines. Access was limited to authorized team members, and all digital files were encrypted. Data will be retained only as long as necessary for the research and then destroyed, safeguarding participants’ privacy throughout the process.

## Findings

Data analysis resulted in five key categories, unified under the main category of ‘Enhancing Integrated and Collaborative Mental Health Care through Overcoming Systemic Barriers and Promoting Patient-Centered Approaches’. The first descriptive category, Challenges of Institutional and Sectoral Boundaries, highlights how legislative and bureaucratic barriers disrupt care continuity in mental health services. The second category, Importance of User-Centered Care and Inclusion, emphasizes the need for early engagement of service users and respect for their preferences throughout treatment. The third category, Interdisciplinary Collaboration and Role Understanding, underscores the importance of clear roles and mutual respect among HP to enhance teamwork. The fourth category, Empowerment and Ownership in Treatment Planning, stresses the significance of involving service users in decision-making to improve outcomes. Finally, the fifth category, Experience and Professional Development, points to the value of continuous learning in enhancing the effectiveness of HP.

### Category 1: Challenges of institutional and sectoral boundaries

HP described how navigating institutional and sectoral boundaries in healthcare posed an ongoing challenge for them. These boundaries often materialize as legislative and bureaucratic hurdles that disrupt the continuity and effectiveness of service user care. Whether transitioning SUs between inpatient facilities, community care, or specialized services, these obstacles are consistently encountered and deeply felt. One nurse highlighted the frustration of dealing with legislative challenges:

Sometimes you run into legislation that another system must handle which can severely hinder continuity in treatment. (Nurse, inpatient unit)

This remark underscores the practical implications of navigating complex healthcare regulations, where delays or disruptions in users care plans can occur. A social worker echoed these sentiments, emphasizing the disjointed nature of healthcare systems:

Healthcare systems are often siloed, with disjointed regulations and practices that do not always align. This lack of cohesion creates significant barriers to seamless mental health care. (Social worker, outpatient clinic)

The social worker’s reflection emphasizes the systemic issues that HPs face, advocating for integrated healthcare policies to facilitate smoother transitions between care settings. Furthermore, a nurse who previously worked in community health succinctly described the bureaucratic challenges:

There are boxes that get in the way. (Nurse, outpatient clinic)

This metaphor vividly portrays the rigid structures and protocols that healthcare professionals must navigate, often at the expense of mental health care efficiency. Another nurse expressed frustration when confronted with these institutional barriers:

It feels like we’re running into roadblocks at every turn. We have the intention to deliver seamless, user-centered care, but the system’s structure makes it nearly impossible to maintain continuity. (Nurse, inpatient unit)

This statement highlights the significant challenges that healthcare professionals face in a fragmented system, where the delivery of mental health care is often disrupted. The quote underscores how institutional and sectoral boundaries, along with bureaucratic and legislative obstacles, impede the continuous care of service users. These challenges emphasize the need for cohesive policies and integrated care models to improve coordination and ultimately enhance outcomes for SU.

### #2: Importance of user-centered care and inclusion

All HPs described finding user-centered care critical in the modern healthcare landscape. This approach emphasizes involving SUs from the very beginning of their care journey, respecting their individual preferences throughout treatment, and ensuring they are well-prepared for discharge. A more detailed discussion on this topic, supported by insights from HPs, highlights the importance of early engagement with SUs. As noted by a seasoned nurse with over 15 years of experience in mental health services, engaging SUs from the start is crucial for effective care.

Involving service users early helps in building trust and ensures that the care plan aligns with their personal health goals and concerns. (Nurse, inpatient unit)

This approach not only empowers SUs but also facilitates a more tailored treatment strategy that can lead to better health outcomes. Respecting SU wishes throughout their treatment process is a fundamental principle of user-centered care. A HP specializing in SU advocacy emphasizes,

It’s not just about treating a condition, it’s about respecting the person dealing with it. Each service users’ values and preferences should guide the decisions we make together. (Assistant nurse, inpatient unit)

This sentiment underscores the importance of collaboration between SUs and healthcare providers in decision-making processes. Careful preparation for discharge is another critical aspect. A hospital discharge planner shared,

We prepare them for when they are going to be discharged. It’s reality. We ensure they understand their medication, follow-up appointments, and any home care requirements. (Nurse, inpatient unit)

This preparation helps prevent readmissions and ensures SUs feel secure and informed as they transition from hospital to home. User-centered care is essential in modern healthcare. It emphasizes early engagement of SU, respects their preferences throughout treatment, and ensures thorough discharge planning. These insights highlight the importance of involving SUs early to build trust and tailor treatment to their individual health goals. Additionally, respecting SUs’ wishes during treatment and ensuring they are well-prepared for discharge are crucial practices for enhancing health outcomes and ensuring a smooth transition from hospital to home.

### # 3: Interdisciplinary collaboration and role understanding

Effective interdisciplinary collaboration in healthcare settings is crucial for delivering high-quality, user-centered care. This type of collaboration requires a deep understanding and respect for the roles and capabilities of various disciplines involved in a person’s care. As one HP explains,

The collaboration is smooth if there’s good understanding among the collaborators. (Nurse, outpatient clinic)

This statement underscores the importance of mutual respect and knowledge among team members, which can significantly reduce conflicts and enhance synergy across sectors. Another key aspect of successful cross sector working is the clarity of roles. Each team needs a clear definition of their responsibilities and the scope of their professional contributions. A nurse practitioner noted,

It’s vital to have clear definitions of the different roles and possibilities available in various places. (Nurse, inpatient unit)

This clarity helps prevent overlap of duties and ensures that each professional’s skills are used effectively, maximizing the benefits for patient care. Additional insights from HP further emphasize these points,

When everyone knows what to expect from each other, it not only streamlines the workflow but also builds a foundation of trust. (Psychomotor Therapist, outpatient clinic)

According to discussions among HP, by fostering an environment where roles are well understood and respected, healthcare teams can improve their effectiveness, leading to better patient outcomes and more efficient use of resources. This approach requires continuous communication, education, and adjustment as roles evolve with advancements in medical practice and technology.

### # 4: Empowerment and ownership in treatment planning

In the realm of mental health services, the significance of empowering SUs by actively involving them in the decision-making process is a central category among HP. This engagement is pivotal for developing personalized and effective care strategies that cater directly to the individual needs of the SUs. As one HP highlighted the essence of empowerment,

Key words are almost SU empowerment. Being met as the person they are. (Nurse, inpatient unit)

This statement underscores the necessity of recognizing and respecting the unique perspectives and experiences of each SU, thereby fostering a sense of ownership and partnership in the treatment process. Another HP emphasized the need for transparency and openness in communications,

Transparency in the decisions we make, being open and honest about the decisions we make. (Social Worker, outpatient clinic)

Such transparency is crucial not only for building trust but also for ensuring that SU are empowered to co-develop their care plans with the support of the professionals around them. This is particularly vital in a cross-sectoral collaborative environment where multiple disciplines and sectors converge to coordinate comprehensive mental health support.

High levels of cross-sectoral collaboration are essential for seamless coordination of mental health services. This involves integrating inputs from various healthcare providers, social services, and possibly educational and occupational sectors, aiming to create a cohesive support system around each SU. As one nurse pointed out,

Cross-sectoral collaboration is not just beneficial but essential in mental health services. It ensures that all aspects of a person’s well-being are addressed comprehensively. (Nurse, inpatient unit)

This integrated approach allows for a holistic view of the SU’s needs, leading to more thorough and coordinated care plans that can significantly enhance treatment outcomes. Furthermore, regular and structured communication channels among different sectors are vital for maintaining the continuity and consistency of care, as emphasized by a social worker,

To truly support our mental health service users, we need robust channels of communication that ensure everyone involved is on the same page. (Social Worker inpatient unit)

By fostering empowerment and ownership among SUs and enhancing cross-sectoral collaboration, mental health services can achieve a higher degree of efficacy and responsiveness. This collaborative model not only respects the autonomy of the individual but also leverages the collective expertise and resources of various sectors, leading to better patient outcomes and a more sustainable health service system.

### # 5: Experience and professional development

The nuanced relationship between a HP’s experience, professional development, and their effectiveness within interdisciplinary and cross-sectoral collaborations is particularly pronounced in the realm of mental health services. Here, the continuous interplay between personal growth and systemic integration plays a pivotal role. The comment from a HP about their transition from an inpatient to an outpatient setting underscores a broader category,

When I came from the inpatient ward, I thought we could handle it all out here because everyone was referred to us. (Nurse, outpatient clinic)

This reflection indicates a shift in perspective that often occurs as professionals engage with varying levels of care and responsibility. It highlights the potential for misconceptions about the scope of services provided in different settings and the importance of adjusting expectations to meet reality. The skills required by professionals for effective for cross-sectoral collaboration in mental health cannot be overstated. Mental health challenges often intersect with various societal and medical factors, and professionals need to work with an appreciation of this. A social worker in mental health elaborates on this integration,

To truly support our mental health patients, we must look beyond the clinical symptoms and consider their social environments, employment status, and family dynamics. (Social Worker inpatient unit)

This statement captures the essence of holistic care, where understanding the full context of a person’s life is as critical as treating their medical symptoms. Further enriching the discussion, a senior nurse comments on the personal and professional growth that arises from interdisciplinary interactions,

Working closely with professionals from different sectors has not only broadened my understanding of patient care but also instilled a deeper respect for the various expertise that each discipline brings. (Nurse, outpatient clinic)

These interactions serve as both a professional challenge and an opportunity, driving personal growth and enhancing the collective capability of healthcare teams to handle complex patient needs effectively.

A Physiotherapist in mental health outreach emphasizes the value of shared experiences and knowledge across sectors,

Each interaction with a colleague from a different sector enriches our approach, allowing us to craft more nuanced and effective interventions for our patients. (Physiotherapist inpatient unit)

This collaborative ethos is essential in mental health, where the intricacies of each case require diverse inputs to ensure comprehensive care.

The reflections of these HP highlight the transformative impact of cross-sectoral collaboration on mental health services. By embracing the complexities of each role and the unique contributions of various sectors, healthcare providers can enhance the quality of care and foster an environment conducive to continuous learning and professional development. This approach not only improves outcomes for patients but also enriches the professional lives of those within the healthcare system, creating a cycle of improvement and innovation.

## Discussion

This discussion explores key categories emerging from the study, focusing on the significance of user-centered care, interdisciplinary collaboration, and the challenges posed by institutional barriers in mental health care. The study reinforces the importance of involving SUs early in the care process, which not only aligns treatment with their personal goals but also enhances trust and efficacy, as highlighted by Davidson (2016) and Biringer et al. (2017). Additionally, the discussion addresses the frustrations related to rigid legislative structures that impede seamless care transitions, echoing the concerns of Söderberg et al. (2022). By connecting these findings to existing literature, this discussion aims to advocate for policy reforms and emphasize the need for continuous professional development to improve cross-sectoral collaboration in mental health services.

The principle of user-centered care is crucial, as highlighted in the study findings and supported by Davidson (2016) and Biringer et al. (2017). These authors emphasize the importance of involving SUs early in their care to ensure treatment plans align with their personal goals, a practice that not only respects individual preferences but also enhances treatment efficacy. This study adds practical examples of how early engagement builds trust and creates effective care plans, echoing the gaps identified by Ness et al. (2014), who advocate for greater patient involvement. Integrating user-centered care within cross-sectoral collaboration frameworks can mitigate challenges associated with disjointed care experiences, as users feel more empowered and engaged throughout their treatment journey. The frustrations concerning institutional barriers, as expressed by study participants, resonate with the issues highlighted by Söderberg et al. (2022). These barriers often stem from rigid legislative and bureaucratic structures that impede fluid care transitions between sectors. The call for streamlined processes and reduced bureaucratic friction underscores a significant area for policy reform. By advocating for legislative changes, healthcare systems can foster a more adaptive environment capable of meeting the dynamic needs of mental health service users, thus responding to calls for integration and flexibility in mental health care delivery (Jørgensen, Lerbæk, et al., 2024).

The mutual respect and understanding among healthcare professionals from various disciplines, as highlighted in this study, support the integrated care models proposed by Jørgensen et al. (2021). This collaborative approach enhances service delivery by ensuring that all participants in the care process from nurses to social workers are aligned in their goals and understand their roles clearly. This alignment is crucial for reducing role ambiguity, which has been identified as a significant stressor and barrier to effective collaboration (Leemeijer & Trappenburg, 2016; Oxelmark et al., 2018).

The insights into empowerment and ownership emphasize the critical role of transparent communication in treatment planning. This aligns with recent healthcare reforms advocating for increased patient autonomy and active participation in decision-making processes (Davidson, 2016; Biringer et al., 2017). The study’s findings illustrate how open communication not only builds trust but also enhances a person’s belief in their treatment, an essential component of successful mental health interventions. Implementing strategies that prioritize transparency can lead to improved SU satisfaction and overall well-being, addressing the holistic needs of SUs in a manner that respects their individuality and preferences (Høgh, 2017). Continuous professional growth is vital for adapting to the complexities of mental health care. The study highlights how exposure to diverse professional contexts enriches HP’ understanding and adaptability. This finding advocates for ongoing education and professional development, ensuring that professionals are equipped with the skills needed to navigate complex collaborative environments effectively. This need aligns with the broader calls for lifelong learning within healthcare professions, facilitating better integration of services and more effective responses to patient needs (Ames et al., 2019).

Building on the foundational work of this study, future research should explore the specific elements of policy reform that can facilitate better cross-sectoral collaboration. Additionally, investigating the long-term outcomes of integrating interdisciplinary respect and collaboration into professional training programs could provide valuable insights. Research focusing on the impact of communication strategies on SU empowerment and treatment adherence could further optimize mental health care practices, enhancing both policy and practical applications.

An essential factor influencing the effectiveness of cross-sectoral collaboration in mental health care is HP morale. High morale among HP’s enhances engagement, communication, and a willingness to collaborate across different sectors. Conversely, low morale can lead to disengagement, poor communication, and resistance to collaborative efforts, ultimately impeding the goals of integrated care. Participants in our study highlighted the significance of morale on collaborative practices. One nurse from an inpatient unit observed that when staff feel supported and valued, it reflects in their work: ‘We’re more open to collaborating and ensuring our patients receive the best care possible’. This sentiment underscores how a positive work environment fosters proactive engagement with colleagues from other sectors. On the other hand, a social worker from an outpatient clinic noted the challenges of working together effectively when the team feels stretched thin and undervalued. These observations align with existing literature, which indicates that staff morale is directly linked to the quality of patient care and the effectiveness of teamwork (Gittell, 2012; Gittell & Hajjar, 2019; Rogers, 2003). High morale contributes to job satisfaction and reduces burnout, leading to better patient outcomes. To improve cross-sectoral collaboration, it is crucial to implement strategies that boost staff morale, such as supportive leadership, recognition of achievements, opportunities for professional development, effective communication, workload management, and inclusive decision-making. By integrating these strategies, organizations can create a supportive environment that not only enhances staff well-being but also improves the effectiveness of cross-sectoral collaboration. This approach ultimately leads to better patient outcomes, as engaged and satisfied staff are more likely to provide high-quality care. Future initiatives aimed at enhancing cross-sectoral collaboration should prioritize boosting staff morale, recognizing the intrinsic link between staff well-being and the quality of patient care.

## Limitations

While this study offers valuable insights into healthcare professionals’ views on cross-sectoral collaboration in mental health care, it is important to interpret the findings with caution due to notable methodological limitations. The reliance on purposive sampling may not fully represent the diverse experiences of professionals, potentially introducing bias into the results. Focusing solely on qualitative data from focus groups risks confirmation bias and may limit understanding of the nuances of interdisciplinary interactions. Additionally, using content analysis alone might not provide a comprehensive framework, suggesting the need for quantitative methods to capture the complexity of collaborative practices. Moreover, new initiatives like pilot projects may exhibit better outcomes not solely because of the effectiveness of the interventions but due to factors such as a fresh and enthusiastic workforce or increased performance resulting from research scrutiny—a phenomenon known as the Hawthorne effect. These factors can temporarily enhance results, making new projects appear more effective than established practices. Therefore, caution should be exercised before implementing widespread changes based solely on pilot study results, as they may overlook systemic challenges that could hinder effective collaboration in the long term. To strengthen future research and enhance practical applications, it is advisable to incorporate more diverse sampling methods, utilize mixed method approaches that combine both qualitative and quantitative data, and adopt a more critical perspective. Further studies with larger samples and longer follow-up periods are necessary to determine the long-term effectiveness and sustainability of these collaborative approaches before applying them broadly. This comprehensive approach will lead to improved strategies for mental health care collaboration and ensure that changes are both effective and sustainable.

## Conclusion

This study explores professionals’ experiences with cross-sectoral collaboration in enhancing mental health care. Key findings highlight the importance of a user-centered approach, where early involvement of service users (SUs) in treatment planning leads to more personalized and effective care. The research identifies significant barriers, such as bureaucratic challenges, that hinder seamless collaboration between sectors, emphasizing the need for policy reforms to improve integration. Interdisciplinary collaboration among healthcare professionals (HPs) is crucial. Mutual respect and clear role definitions enhance teamwork and service delivery, while transparent communication empowers SUs and improves treatment adherence. Overall, the study underscores the necessity for improved collaboration, policy changes, and ongoing professional development to create more effective and integrated mental health services. To enhance mutual understanding and reduce misconceptions between different sectors in mental health care, we recommend implementing periodic job swaps among healthcare professionals. This practical strategy allows staff to experience each other’s roles firsthand, fostering empathy and improving collaboration. By gaining insights into the challenges and workflows of colleagues in other sectors, professionals can develop a more cohesive approach to patient care. Implementing job swaps can lead to more integrated services and ultimately improve outcomes for service users by ensuring that all team members are aligned in their efforts to provide holistic, patient-centered care.
